# Two-photon-induced stretchable graphene supercapacitors

**DOI:** 10.1038/s41598-018-30194-2

**Published:** 2018-08-06

**Authors:** Litty V. Thekkekara, Xi Chen, Min Gu

**Affiliations:** Laboratory of Artificial-Intelligence Nanophotonics, School of Science, RMIT University, Melbourne, Victoria, 3001 Australia

## Abstract

Direct laser writing with an ultrashort laser beam pulses has emerged as a cost-effective single step technology for realizing high spatial resolution features of three-dimensional structures in confined footprints with potential for large area fabrication. Here we present the two-photon direct laser writing technology to develop high-performance stretchable biomimetic three-dimensional micro-supercapacitors with the fractal electrode distance down to 1 µm. With multilayered graphene oxide films, we show the charge transfer capability enhanced by order of 10^2^ while the energy storage density exceeds the results in current lithium-ion batteries. The stretchability and the volumetric capacitance are increased to 150% and 86 mF/cm^3^ (0.181 mF/cm^2^), respectively. This additive nanofabrication method is highly desirable for the development of self-sustainable stretchable energy storage integrated with wearable technologies. The flexible and stretchable energy storage with a high energy density opens the new opportunity for on-chip sensing, imaging, and monitoring.

## Introduction

Energy storage demands for the technological advancements in micro-stretchable, wearable, and portable applications^1^ have enhanced the requirement of self-powered and self-sustaining systems which need to be supported by an integrable high-performance energy storage in confined footprints and light weights^2^. The development of on-chip in-plane micro-supercapacitors (MSCs) using laser scribed graphene (LSG) has attracted tremendous attention due to the integration possibilities with the small-scale devices^3^. However, the deliverable aerial capacitance from that MSCs^4^ is minimal for powering micro-devices which limit the potential of autonomous systems.

To address this challenge, three-dimensional (3D) energy storages have emerged as a new concept which can potentially enable the miniaturization and integrability in a single platform without losing the performance of energy storages by the effective utilization of the volumetric capacitance performance^5^. However, the current research in this area has been mainly focused on the use of pseudocapacitive materials^6^ and gold nanoparticles for LSG^7^, which makes those MSCs lacking the stretchability^8^ essential for wearable applications^9^ due to the fragile nature of gold. Although direct laser writing (DLW) with ultrashot femtosecond (fs) laser beams has been adopted to generate 3D MSCs^6–8^, two-photon excitation induced in the focus of a high-numerical objective has not been explored. Due to the 3D confinement in the tight focus^10^, this technology has opened the enormous potential for the industrial scale production^11^. As a result, this single-step fabrication technique has generated various complex 3D and free-standing structures at micron and sub-micron scales in telecommunications^12^, biomedical applications^13^, free-form optics^14^ and displays^15^.

**Figure 1:**
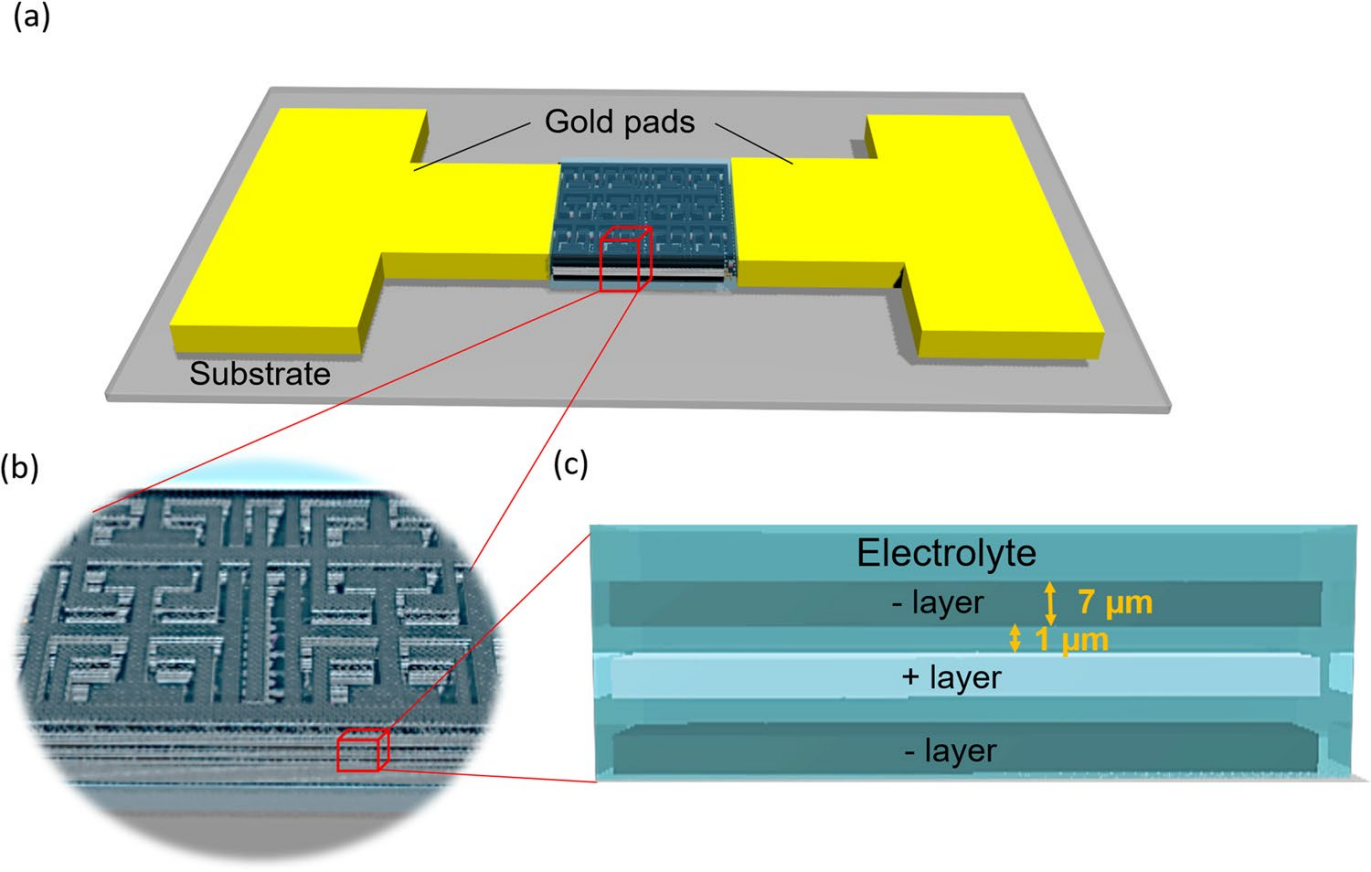
Two-photon-induced (2PI) 3D graphene micro-supercapacitor (MSC) using a femtosecond (fs)-laser beam. (**a**) Schematic of 3D 2PI-graphene MSC inspired by *Fern leafs* using a fs-laser beam with the gold pads as current collectors. (**b**) Highlighted view of the 3D 2PI-graphene MSC. (**c**) Cross-sectional view of the alternate layers of positive and negative 2PI-graphene with an interlayer spacing of ~1 µm.

In this paper, we demonstrate the fabrication of a bioinspired 3D two-photon-induced (2PI)-graphene MSC directly on an elastomeric polydimethylsiloxane (PDMS) substrate, using the two-photon DLW approach to generate the electrode inter-distance down to 1 mm (Fig. 1(a)) in 40 minutes of fabrication time. A detailed flowchart of the fabrication process for 3D 2PI-graphene MSC is included as Fig. S1. One of the commonly used techniques for MSC fabrication is laser writing using continuous wave (cw) which limits the spatial resolution inturn leading to the larger interelectrode distances^4^. On the other hand, the MSCs fabricated using electron beam lithography (EBL) limits the possibilities of extension to three-dimensional which inturn affects the capacitance^16^ in limited aerial footprints.

The adoption of PDMS substrate, in addition, allows us to achieve the stretchable feature. Our result shows the charge transfer capability enhanced by order of 10^2^ and the energy storage density exceeding the current lithium-ion batteries. More importantly, the stretchability and the volumetric capacitance reach 150% and 86 mF/cm^3^ (0.181 mF/cm^2^), respectively, which are enhanced by more than three times and 100 times compared with the state of the art results^17^.

## Results and Discussions

### 3D two-photon-induced electrodes

The 2PI multilayered electrode structure is achieved with the 3D extension of the two-dimensional structure based on the fractal pattern derived from fern tree leaves^4^ (Fig. 1(b)) with interconnected electrode layers separated by electrolyte layers as shown in Fig. 1(c). The fractal pattern self-repeating pattern is adopted for the electrodes due to the high surface area achievement in comparison to other electrode designs^4^. We can construct the space filling curve^4^ for 3D 2PI-MSC by iterating a 2D space-filling curve described in the earlier literature^18^ through a layer by layer approach. Space-filling curve designs follow the linear equations in the iteration with a dimensionality represented by the Hausdorff dimension (HD)^19^ which is the measure of the local size of a set of numbers and can be calculated by using the Box-counting method^20^. The relationship between HD, the linear scaling (L) and the resulting increase in size (S) which is the number of self-similar units generated after scaling for a layer by layer plane construction of 3D object can be written as below

1 $$D={\mathrm{lim}}_{L\to 0}\,\mathrm{log}(S)/\,\mathrm{log}(L)$$

The optimization of such a 3D 2PI-graphene electrode structure could be calculated from the HD for the fractal electrodes through the consideration of the electrode width, d_1_ for a total 2PI-graphene film thickness of 23 µm, as shown in Fig. 1(c).

We use a fs-laser beam to achieve the multilayer graphene oxide (MLGO) reduction for the fabrication of a 3D 2PI-graphene MSC in a footprint of 1 mm^2^. Beyond the miniaturization aspect, the high spatial resolution of the focused fs-laser beams can lead to the theoretically predicted ideal inter-electrode distance below 25 µm in the MSC for the optimum electrolyte ionic path^21^.

It has been demonstrated that the microstructures fabricated by fs pulses are mainly a non-thermal fabrication process^15,22^, which involve the multiphoton absorption within the time scales of less than picosecond resulting in the suppression of heat diffusion and the elimination of the heat affected zone. As a result, this fabrication method can lead to structures with high-resolution. However, the graphene oxides photoreduction at a wavelength of 800 nm may be contributed by a combination of photothermal and photochemical^23^ assisted with shock waves as the pulse repetition rate becomes higher. In order to understand the phenomenon in detail, we conducted a detailed analysis of the photoreduction of graphene oxide film under different repetition rates of 80 MHz, and 10 kHz which can be found in Supplementary Notes and the best conditions obtained under 80 MHz is considered for the fabrication of 2PI-graphene MSCs.

The two-photon absorption behavior in the photoreduction of graphene oxides is studied using the standard method of photoluminescence (PL) measurements^24^. The PL measurements in single-layer graphene oxide film as a function of the laser fluence under a repetition rate of 80 MHz confirms the two-photon absorption (Fig. S2) during the photoreduction process which, in turn, leads to the temperature rise in the process (Fig. S3). The temperature rise during the photoreduction process was calculated using the numerical simulations based on heat balance equations with the Cosmol Multiphysics software (Supplementary Note)^25^.

The 2PI phenomena lead to polycrystalline nature for the obtained 2PI-graphene film as observed from the transmission electron microscopy (TEM) image (Fig. S4). In addition, from Table S1 we can find that the material reduction properties of the 2PI-graphene film have been improved with an uniform porous nature for the 2PI-graphene film in comparison to the repetition rate of 10 kHz (Fig. S5).

### Multilayer 2PI-graphene film optimization

For the improvement of the MSC energy storage density, the thickness of the graphene oxide film is a significant factor. In our studies, we observe that the focused laser beam with an objective of NA 1.4 generates the stronger thickness reduction in the 2PI-graphene film compared with an objective of NA 0.6 (Fig. S6). The optimized holey graphene oxides photoreduction condition (Fig. S6, Supporting Information) is used to engineer the multilayer structures prepared by the layer by layer assembly of positive and negative graphene oxide films (see Experimental Section) with a thickness of 42 µm for three-layer graphene oxides. Raman spectra of positive (ammonium functionalized) and negative graphene oxides were given in Fig. S7(a). The I_D_/I_G_ ratios obtained were 1.39 and 1.08 for positive and negative graphene oxides respectively which indicates the increase of defects in the positive graphene oxide layers. The Raman spectra of MLGO were given in Fig. S7(b) from which I_D_/I_G_ ratio was calculated to be 1.26.

The use of the lower repetition rate of 10 kHz results in the higher oxygen group content in the 2PI-graphene structure (Fig. S8). Based on these observations, we use the repetition rate of 80 MHz with the 0.6 NA 20x objective for the fabrication of MSCs with a layer distance of 1 µm achieved by the DLW setup from *Nanoscribe* with a pulse width of 100 fs. The temperature involved in the reduction process of graphene oxides using 80 MHz is calculated to be 800 °C (Fig. S3).

In order to confirm the two-photon absorption behavior for the MLGO reduction during the fs-laser beam irridation, we plot the PL intensity versus the laser fluence on a logarithmic scale, as shown in Fig. S9(a) for a three-layer and a two-layer 2PI-graphene film (Fig. S9(b)). The slope obtained from the plots is 2.08, and 2.13 for the 2PI-graphene film, similar to the single-layer GO film reduction (Fig. S2).

**Figure 2:**
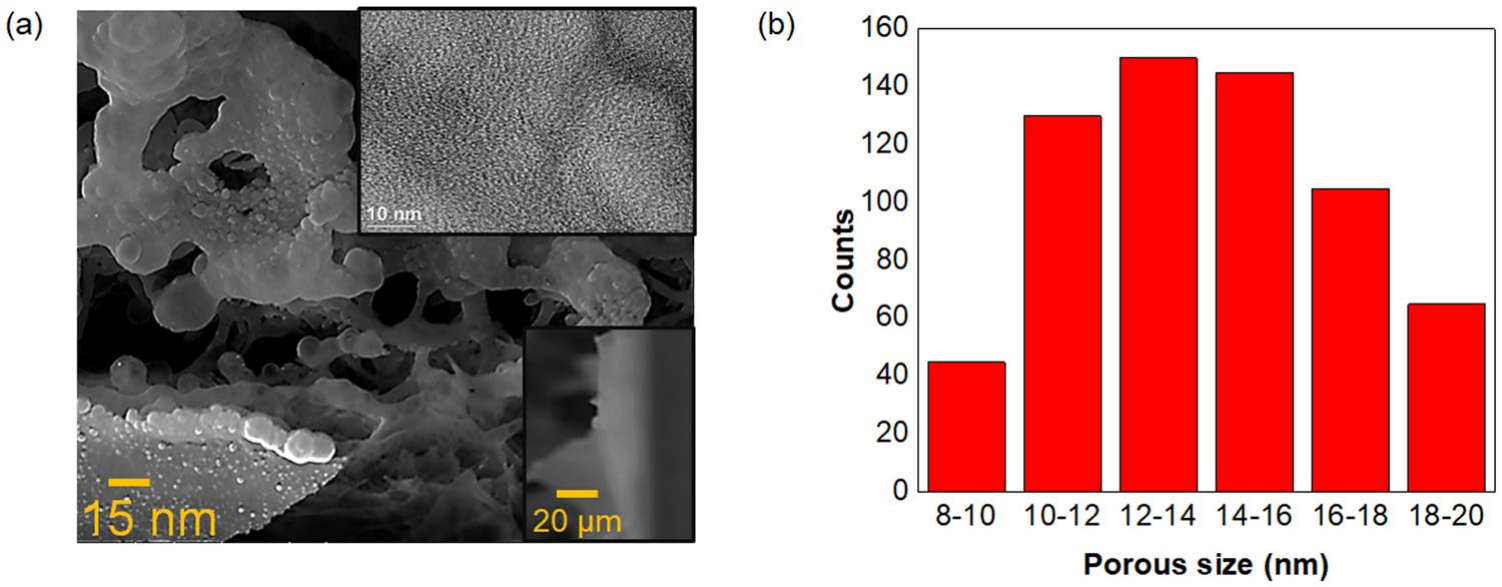
Characterizations for the 3D 2PI-graphene film. (**a**) SEM image of the polycrystalline 3D 2PI-graphene film at a pulse repetition rate of 80 MHz for the wavelength of 800 nm. Highlighted: High-resolution TEM image of the 2PI-graphene film (right top). SEM cross-section for the thickness of the 2PI-graphene film (right bottom). (**b**) Porous size distribution within 2PI-graphene films obtained using 800 nm wavelength, 80 MHz repetition rate femtosecond laser beam and 0.6 NA.

The 2PI temperature rises observed in the reduction process in the 3D 2PI-graphene film (Fig. 2(a)) leads to a porous structure in the film with a pore distribution calculated from the scanning electron microscopy (SEM) images using the *Image J* software^26^ (Fig. 2(b)). The film has a pore size range from 8 to 20 nm with a polycrystalline nature observed from TEM (Fig. 2(a)) measurements. The porosity of the film are further confirmed using Barnett-Joyner-Halenda (BJH) analysis^27^ (Fig. S10) and small angle scattering (SAXS) measurments^28^ (Fig. S11). The formation of defects and sp^2^ crystalline size for various layers of 2PI-graphenes are studied using Raman and X-ray photoelectron spectroscopy (XPS) spectroscopies (Table S1). We can observe an increase in the number of defects^29^ from the Raman spectra of different layers of the graphene oxide film (Fig. S12) along with an improved sp^2^/sp^3^ ratio in the 3D 2PI-graphene film structure under the optimum fs-laser beam irradiation conditions.

**Figure 3:**
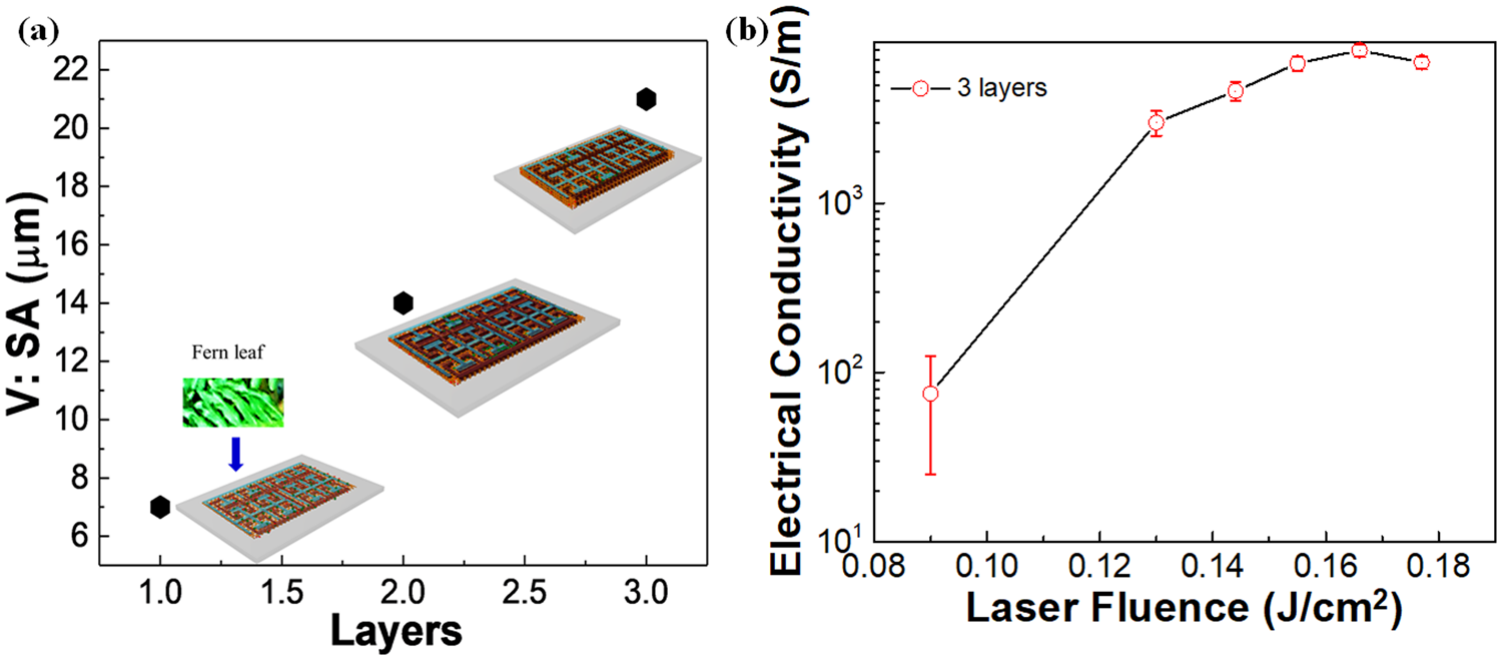
Surface area and electrical property of 3D 2PI-graphene film. (**a**) The plot for the volume to surface area ratio, V: SA, for a different number of layers of the 2PI-graphene MSC. (**b**) The electrical conductivity obtained for the 3D 2PI-graphene film with error bars given in red.

The electrical conductivity of the MSC electrodes determines the ability to hold the electrolyte ions within the pores and enable the faster ion transport during the charge-discharge process^30,31^. The volume to the surface ratio used for the optimization of the 3D 2PI-graphene MSCs (Fig. 3(a)) is calculated by considering an average pore size of 12 nm obtained from Figs 2(a), S10, S11. The minimum electrode width, d_1_ that can be attained based on the maximum electrical conductivity for the 2PI-graphene film (Fig. 3(b)). The obtained electrical conductivity for single-layer and two-layers of graphene oxide film is given in Fig. S13. The relevant procedure is explained as follows.

The area of one pore, *A*_*pore*_ is given as

2 $${Area}\,{of}\,{one}\,{pore},\,{Apore}=\pi {r}^{2}$$

Using this equation, we can calculate the number of pores per square micrometer,

3 $$Npore/{\mu }{m}^{2}={A}_{pore}\times {N}_{pore}$$

Based on this calculation, we can further calculate the effective electrode active area; *Active area*_*eff*_ as given by

4 $$Active\,are{a}_{eff}=Npore/{\mu }{m}^{2}\times Active\,are{a}_{geo}$$

The useful active electrode volume, *Active volume*_*eff*_ otherwise V_*3D 2PI*_ can be calculated by considering the thickness, *t* of the LSG and the effective active area, *Active area*_*eff*_:

5 $${{\rm{V}}}_{{3D}{2PI}}=Active\,are{a}_{eff}\times t$$

The ratio of the volume to surface area, *V*: *SA* in µm is calculated (Fig. 3(a)) as follows:

6 $$V:SA={{\rm{V}}}_{{3D}{2PI}}/Active\,are{a}_{eff}$$

As a demonstration, we consider the three-layer graphene film for the 2PI-graphene MSC fabrication.

### Stretchable 3D 2PI-graphene MSCs

Based on the understanding of the phenomenon behind the reduction of the graphene oxide film, the 3D stretchable 2PI-graphene MSCs are fabricated. The resultant 3D 2PI-graphene MSC (Fig. 1(c)) has a layered electrode thickness of 21 µm with an interlayer spacing of 1 µm and an individual electrode width of 4 µm in an area of 1 mm^2^. The interdistance between the electrodes is 1 µm, as given in Fig. S14 with a median porous electrode size of 20 nm (Fig. 2(a)). The stretchable and non-stretchable properties of the obtained 3D 2PI-graphene MSCs which are directly written on the PDMS substrate can be analyzed using the electrochemical measurements including cyclic voltammetry (CV) and galvanostatic charge/discharge (CC) with a Keithley ECE 2400 source meter with two tungsten probes of diameter 5 µm.

**Figure 4:**
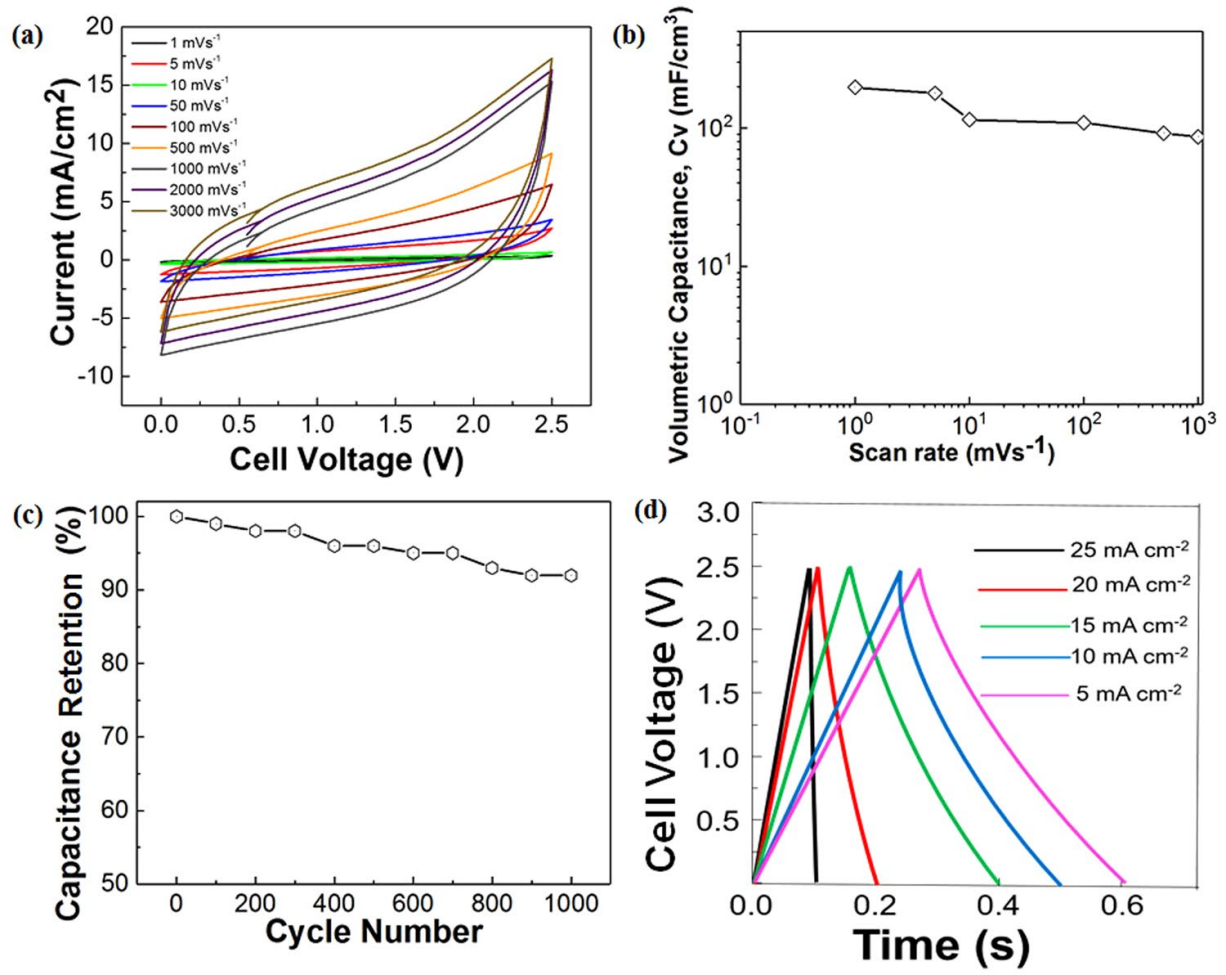
Electrochemical characterizations on the 3D 2PI-graphene MSC. (**a**) Average CV curves for 10 cycles of the 3D 2PI-graphene MSC at different scan rates. (**b**) The volumetric capacitance of the 3D 2PI-graphene MSC calculated from the CV plots. (**c**) Capacitance retention of the 3D 2PI-graphene MSC obtained from the CV measurement up to 1,000 cycles for a scan rate of 1,000 mV s^−1^. (**d**) CC curves for 10 cycles of the 3D 2PI-graphene MSC at different charge densities.

The electrochemical performance of the 3D 2PI-graphene MSC under the non-stretchable condition is given in Fig. 4. The CV measurements conducted at a scan rate of 5 to 3,000 mV s^−1^ with a step size of 0.1 V (Fig. 4(a)). It was observed that a volumetric capacitance of 196 mF cm^−3^ is obtained at lower scan rates and a volumetric capacitance of 86 mF cm^−3^ for a scan rate of 1000 mV s^−1^ (Fig. 4(b)). A capacitance retention of almost 90% at a scan rate of 1,000 mV s^−1^ is obtained for 1,000 cycles (Fig. 4(c)), and the CC measurements show a nearly triangular behavior with an internal drop of 0.1 V (Fig. 4(d)). These factors have resulted in a two-fold increase of the rate transfer capabilities (power density) and an energy density of 10^−1^ Wh cm^−3^ which exceed the lithium-ion batteries at the lower scan rates (Fig. S15). A performance plot for the energy and power densities based on the areal capacitance of this work can be found in Fig. S16. To avoid the influence of gold pads on the performance of 3D 2PI-graphene MSCs, the energy and the power densities are calculated with the removal of the effect of gold pads and the graphene oxide material (See Methods).

The first physical mechanism for the improvement of the 3D 2PI-graphene MSC performance is the increase in the electrode volume in the given surface area. The second mechanism for the improvement is contributed from the defect induced 3D 2PI-graphene films which lead to the domination of the radial or edge diffusion in the electrolyte ion flow^32^ between the 2PI-graphene electrode layers for the miniaturized energy storages which leads to the consideration of a parallel arrangement of capacitances (Eqn. 7) along the three layers of 2PI-graphene electrode as shown in Fig. 1(c).

7 $${\rm{C}}={{\rm{C}}}_{1}+{{\rm{C}}}_{2}+{{\rm{C}}}_{3}$$

The energy density from 3D 2PI-graphene MSCs is less than the effective parallel capacitance contributed from the three layers of the electrode due to the difference of defects in the positive and negative GO film used for the reduction. The growth of defects with the increase of layers, in turn, facilitates the improved amount of pores within the 3D 2PI-graphene film which also contributes to the performance enhancement in these miniaturized MSCs with ionic gel. A comparative table for the performance of single layer 2PI-graphene MSCs and two-layer 2PI-graphene MSCs are given in Table. S2.

**Figure 5:**
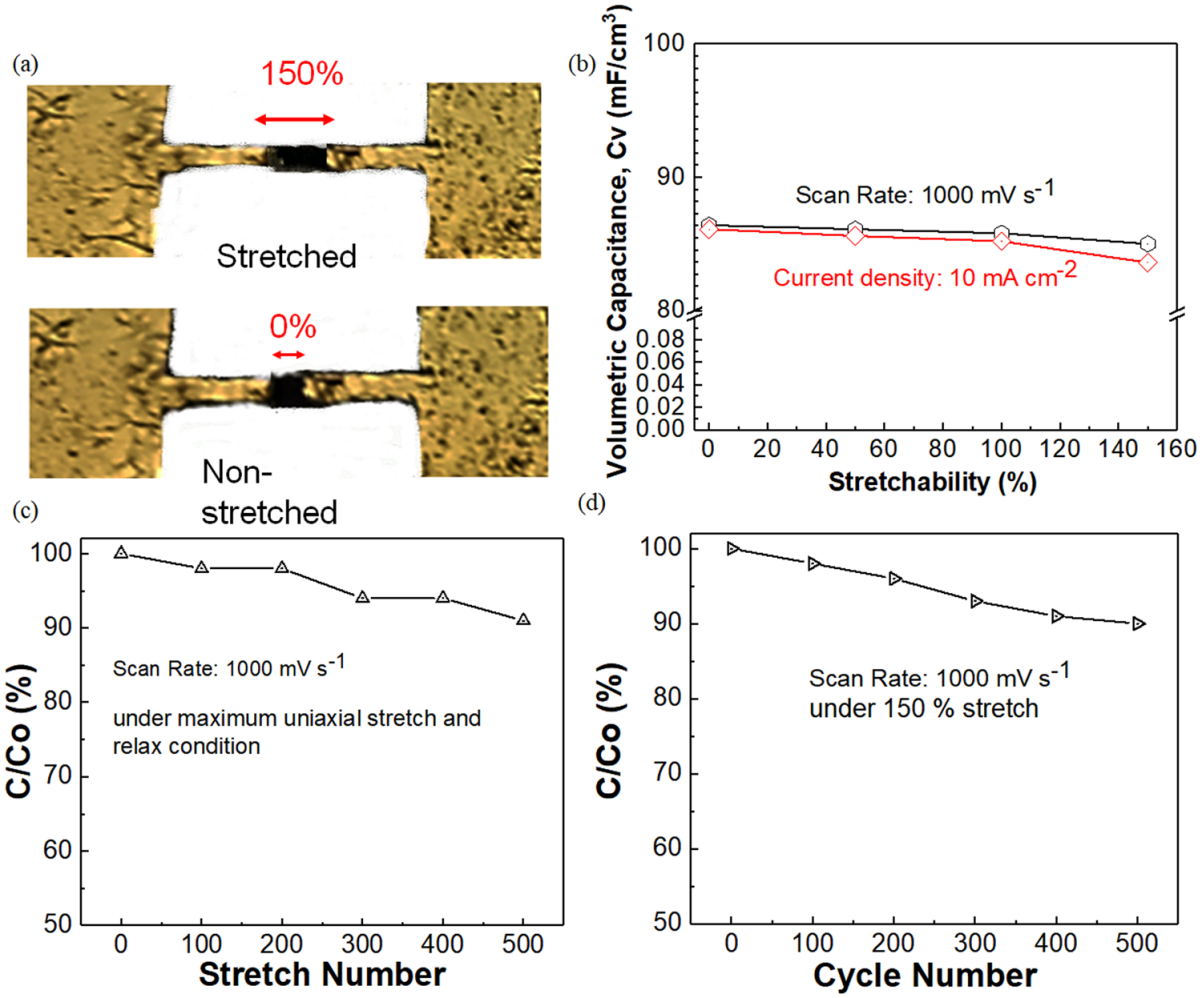
Characterization of stretchable 3D 2PI-graphene MSC. (**a**) Image of the stretched and non-stretched 3D 2PI-graphene MSC. (**b**) Volumetric capacitance calculated for stretchability from 0 to 150% measured using CV for a scan rate of 1,000 mV s^−1^ and using CC for a current density of 10 mA cm^−2^. (**c**) Capacitance retention of the 3D 2PI-graphene MSC obtained from the CV measurement up to 500 cycles for a scan rate of 1,000 mV s^−1^ and a repeating number of stretching and relaxing along the uniaxial direction. (**d**) Capacitance retention of the 3D 2PI-graphene MSC obtained from the CV measurement up to 500 cycles for a scan rate of 1,000 mV s^−1^ with the maximum stretchability of 150%. All data provided are an average of 10 repeated cycles.

The all-solid-state stretchable 3D 2PI-graphene MSCs (Fig. 5(a)) using ionic gel ([BMIM][NTF_2_]) shows an almost quasi-rectangular behavior upto a 150% stretchability as given in Fig. S17(a) for the CV measurements at a scan rate of 1,000 mV s^−1^ with a step size of 0.1 mV and the obtained volumetric capacitance is around 86 mF cm^−3^ (Fig. 5(b)). CC measurements conducted for different stretch distances under a current density of 10 mA cm^−2^ shows triangular shape maintenance with an internal drop of 0.1 V (Fig. S17(b)) with a volumetric capacitance up to 86.1 mF cm^−3^ (Fig. 5(b)). The capacitance retention of 90% can be seen in Fig. 5(c) for the repetition of uniaxial stretching and relaxing condition up to 150%. Figure 5(d) shows capacitance retention up to 90% for the CV measurement cycles up to 500 under a maximum stretchable condition of 150%. The self-healing property of the 3D 2PI-graphene film contributes to the 200% enhanced stretchability with a reduction of 99.7% in MSC area compared to other LSG supercapacitors obtained from polyamide^17^ (Table S3) due to the excellent reversibility of the GOs performance^33^. All the reported measurements are an average data of 10 repeated cycles.

## Conclusion

In summary, we have demonstrated the fabrication of a stretchable 3D 2PI-graphene MSC with the electrode distance down to 1 µm, a volumetric capacitance of 196 mF cm^−3^ at low scan rates and a stretchability up to 150% using the fs-laser beam. The further reduction in the electrode distance below 1 mm should be possible if 3D superresolution nanofabrication is adopted^34^, which could increase the energy density significantly. A large industrial scale MSC fabrication can be achieved with the use of advanced DLW techniques based on parallel 3D multifocal array generation^35^. The cost-effective on-chip flexible and stretchable energy storages^36^ will find applications in wearables^37^ and flexible devices like e-skin^38^ and artificial intelligence devices like memristors^39^.

## Methods

### Fabrication of 3D 2PI-graphene MSCs

We conducted the photoreduction process of single-layer graphene oxide reduction using laser irradiation under the repetition rates of 80 MHz and 10 kHz at a wavelength of 800 nm from a Chameleon OPO laser. 3D 2PI-graphene MSCs of area 1 mm^2^ and thickness 23 µm were fabricated in the graphene oxide film of thickness 44 µm on a polydimethylsiloxane (PDMS) substrate by using a commercial direct writing setup *Nanoscribe* which has a wavelength of 800 nm, pulse width 120 fs and repetition rate of 80 MHz The PDMS substrate (Sylgard 184, Dow Corning,) was prepared in a mixing ratio of 10:1. The air was evacuated from the PDMS mixture using the vacuum. The polymer was crosslinked at 80 °C for one hour in a convection oven. The laser fluence used for writing the graphene oxide film was 0.18 J/cm^2^ with a 20x objective of 0.6 NA. The fabrication of 2PI-graphene MSCs was followed by filling the MSCs with electrolyte ionic gel and allowed to dry overnight. (Refer: Fig. S1)

### Preparation of multilayer graphene oxide films

Graphene oxide films were prepared through the layer by layer assembly using the electrostatic attraction between the positive and negative graphene oxide layers. Hummer’s method was used to synthesize the negatively charged graphene oxides (Sigma-Aldrich)^40^. The positively charged graphene oxide film was prepared using the ammonium functionalization (Sigma-Aldrich)^41^. 1.3 mg/ml of each graphene oxides were dispersed in water and drop cast on a glass substrate. Each layer of graphene oxides was dried at 60 °C, and the process was repeated until the desired number of alternate layers of graphene oxides (MLGO) was achieved.

### Preparation of electrolytes

We used an ionic gel electrolyte. The ionogel was prepared by mixing fumed silica nanopowder (7 nm in size, Sigma-Aldrich) with the ionic liquid (1-butyl-3-methylimidazolium bis (trifluoromethyl sulfonyl) imide [BMIM] [NTf_2_] (Sigma-Aldrich) in a ratio of 0.03 g/1.0 g and the mixture was stirred under a nitrogen atmosphere for about 5 hours. The electrochemical window of the electrolyte is 2.5 V.

### Characterization

Raman spectra were obtained for different layers of graphene oxides using a Renishaw Raman spectrometer equipped with an Nd-YAG laser beam of wavelength 514 nm. Scans were performed between 1,000 and 2,700 wavenumbers with a laser spot size of 1 µm. The background corrected spectrum was normalized by dividing the data by the maximum intensity. The XPS measurements were conducted using the Thermo K-alpha X-ray Photoelectron Spectrometer (XPS) using monochromatic, Micro-focused Al K-α source.

SEM images were conducted using Philips XL30 SEM with a vacuum of 10^−9^ Torr and TEM measurements were conducted using the JEOL 2010 with samples fixed on a copper grid. Porosity measurements of the obtained film is determined using three methods: the diameter analysis of the SEM images using Image J software, BJH method using Surface Area and Porosity Analyser (Tristar; Micromeretics) and Small-angle-X-ray scattering (SAXS) measurements. SAXS was conducted on a microcalix SAXS system (Bruker) using Cu Ka radiation (50 kV, 10 mA). The sample was placed into a 1.5 mm outer diameter quartz capillary. Background measurement was on an empty capillary. Measurements were carried out under vacuum at 20 °C with an exposure time of 2 hours. Primary masking, data reduction and background subtraction and were carried out in BioXTAS Raw^42^. The pores were modeled as a collection of polydisperse spheres of air within the 2PI graphene matrix^28^, and fitting was carried out in SasView^43^ using a standard model^44^ with a Gaussian distribution. The best fit yielded an average radius of 18.7 nm with a polydispersity of 14.6%.

Two-point conductivity measurements on 2PI-graphene films reduced under different repetition rates were conducted using the Keithley 2400 series source meter wired through two micro-positioners using tungsten probes of tip diameter 5 µm. Measurements were taken by varying the applied voltage between 0.1 V to 4.5 V. The rectangle patterns of area 300 µm × 300 µm were connected to gold electrical pads on each side. The electrical conductivity study was performed by keeping the probes on each side of gold electrical pads. With the thickness information, the bulk conductivity was evaluated using the following equation:

8 $$\sigma =l/RA\,$$

The electrochemical measurements were undertaken using the Keithley ECE 2400 source meter with a tungsten probe of diameter 5 µm and an exposed area 2.25 mm^2^ as the current collectors. Open-circuit measurements were performed initially for about one hour before the electrochemical tests to ensure the stable conditions. We used an ionic gel electrolyte to study the performance of obtained 3D 2PI-MSCs under stretched and non-stretched conditions. The CV measurements with a step size of 0.1 mV from 0 to 2.5 V at a scan rate of 1–3,000 mV s^−1^ with an average of 10 cycles.

The capacitance was calculated from the CC curves at different current densities using the formula^21^,

9 $${{\rm{C}}}_{{\rm{device}}}={\rm{i}}/(-{\rm{dV}}/{\rm{dt}})$$

where *i* is the applied current (A), and *dV/dt* is the slope of the discharge curve (in volts per second, V/s).

The energy and power densities of the 3D 2PI-graphene MSCs were calculated by taking away the effect of the capacitance from the gold pad, and MLGO film^25^. The volume specific capacitance was calculated using the formula,

10 $${{\rm{C}}}_{{\rm{volume}}}=({{\rm{C}}}_{{\rm{total}}}-{{\rm{C}}}_{({\rm{Gold}}+{\rm{MLGO}})})/{{\rm{V}}}_{3{\rm{D}}2{\rm{PI}}}$$

where C_total_ is the total capacitance obtained from the 3D 2PI-graphene electrode connected with the gold probe electrodes, C _(Gold+MLGO)_ is the capacitance contributed by the gold and MLGO film separator, V_3D 2PI_ is the volume of the 3D 2PI-graphene MSCs.

The energy density, E_den,_ and the power density, P_den_ (rate of charge transfer) were calculated as the following:

11 $${{\rm{E}}}_{{\rm{den}}}={{\rm{E}}}_{{\rm{total}}}$$

12 $${{\rm{P}}}_{{\rm{den}}}={{\rm{P}}}_{{\rm{total}}}-{{\rm{P}}}_{({\rm{Gold}}+{\rm{MLGO}})}$$

Total energy, E_total,_ and P_total_ of the device can be calculated by the formula:

13 $${{\rm{E}}}_{{\rm{total}}}={{\rm{C}}}_{{\rm{v}}}\times {({\rm{\Delta }}{\rm{E}})}^{2}/(2\times 3600)$$

14 $${{\rm{P}}}_{{\rm{total}}}={\rm{\Delta }}{\rm{E}}/4{{\rm{R}}}_{{\rm{ESR}}}\times {{\rm{V}}}_{3{\rm{D}}2{\rm{PI}}}$$

where E_total_ is the energy density in *Wh/cm*^3^, *Cv* is the volumetric capacitance, ΔE is the operating voltage window in Volts, R_ESR_ is internal voltage drop at the beginning of the discharge, *V*_*drop*_, at a constant current density (*i*) calculated from the CC measurements and given as

15 $${{\rm{R}}}_{{\rm{ESR}}}={{\rm{V}}}_{{\rm{drop}}}/2{\rm{i}}$$

## Electronic supplementary material

Supplementary Information
