# Supplementary material for: Two-photon-induced stretchable graphene supercapacitors
Source: Sci Rep. 2018 Aug 6;8:11722. doi: 10.1038/s41598-018-30194-2 (PMC6079041; doi:10.1038/s41598-018-30194-2)
Supplement: Supplementary file 1 — Supplementary Information [file 41598_2018_30194_MOESM1_ESM.docx]

Supplementary Materials for

Two-photon-induced stretchable graphene supercapacitors

Litty V. Thekkekara, Xi Chen, Min Gu*

*Correspondence to: [min.gu@rmit.edu.au](mailto:min.gu@rmit.edu.au)

**This File includes**

Supplementary Notes

Figs. S1 to S17

Tables. S1 to S3

**Supplementary Notes**

**Photoreduction of the single-layer graphene oxide film**

For the single-layer graphene oxide film, we performed photoluminescence (PL) measurements under excitation at a wavelength of 800 nm for the 80 MHz repetition rate using 100x with 1.4 NA oil and 20x with 0.6 NA objectives. The slopes of the log-log plots of the laser fluence versus photoluminescence (PL) vary from 2.01 to 2.02 (Fig. S2), which confirms that the graphene oxides photoreduction involves two-photon absorption and is similar to the reported two-photon absorption in graphene oxide nanoparticles using a femtosecond (fs)-laser beam^1^.

To understand the influence of photothermal mechanism^2^ under irradiation by a 80 MHz fs-gaussian pulse train, we made an analytical model consisting of 40 laser beam pulses interacting with multilayer GO (MLGO) film based on the heat balance equations given in the reference^3^ using a finite element method (FEM) (*Comsol Multiphysics* software). It is observed that the temperature increases with the number of graphene oxide layer up to 800 *◦*C, as shown in Fig. S3.

We can observe a polycrystalline behavior for the two-photon induced (2PI) graphene films from the transmission electron microscopy (TEM) image due to the formation of smaller crystalline sizes (Fig. S4). An equal pore distribution for the 2PI-graphene film is observed with the repetition rate 80 MHz (Fig. S5 (a)) while the lattice disorders are irregular as the repetition rate goes low from the scanning electron microscopy (SEM) images (Fig. S5 (b)).

The most common phenomenon observed in the obtained 2PI-graphene films is the reduction in the thickness after the direct laser writing process. The focused ion beam (FIB) method is used to obtain a cross-section of the reduced region in the 2PI-graphene film obtained with a repetition rate of 80 MHz using a 100x oil objective with NA 1.4 and a 20x air objective with NA 0.6 (Fig. S6). The thickness reduction observed for the obtained 2PI-graphene film reduction using a 100x objective is higher by a factor of 2 (Fig. S6 (a)) than a 20x objective, due to the impact of tightly focused light interaction on the material^4^ as shown in Fig. S6 (b).

A detailed investigation regarding the deoxygenation in the obtained 2PI-graphene films is further made using the standard methods of X-ray photoelectron spectroscopy (XPS) by calculating the sp^2^/sp^3^ ratio and the I_D_/I_G_ ratios from the Raman analysis, as shown in Table 1 under the optimum laser fluence of 0.18 J/cm^2^. The synthesized graphene oxide film has two distinct Raman peaks-D band located around 1331 cm^-1^ and the G band located around 1585 cm^-15^. The D band is related to the sp^3^ carbon bonds as well as the defect formation, and the G band usually indicates the sp^2^ carbon bonds in the graphene oxide film. An increase of I_D_/I_G_ to 0.99 indicates the gradual change from sp^3^ to sp^2^ states in the2PI-graphene film which is further justified by XPS results in the sp^2^/sp^3^ ratio of 1.6.

The crystalline sizes of the C-C domains in the plane (L_a_) are calculated to be 18 nm to 16 nm for the2PI-graphene film obtained with the repetition rate 80 MHz from the ratio of the integrated intensity of the G peak (I_G_) and D peak (I_D_) using the formula given in reference^6^,

L = 2.4 x 10^-10^ x λ^4^/ (I_G_/I_D_) (S1)

where (*λ*) is the wavelength of the Raman laser (514 nm).

From the XPS spectra analysis of the 2PI-graphene films reduced using the repetition rates of 80 MHz (Fig. S8(a)) and 10 kHz (Fig. S8(b)), the residual oxygen groups are observed which confirms that the pulse peak power energy using fs-laser beam irradiation is not sufficient enough to remove the oxygen groups completely from the graphene oxide structure.

The electrical conductivity measurements using the two-probe method with a Keithley source meter 2400 shows an order of 10^3^ S/m with the repetition rate 80 MHz. The increase of defects can result in the smaller discontinuous C-C cluster formation which leads to the decrease in electrical conductivity due to the disturbed electric charges (Fig. S11) compared with the pristine graphene.^5^

**Supplementary Figures**

**
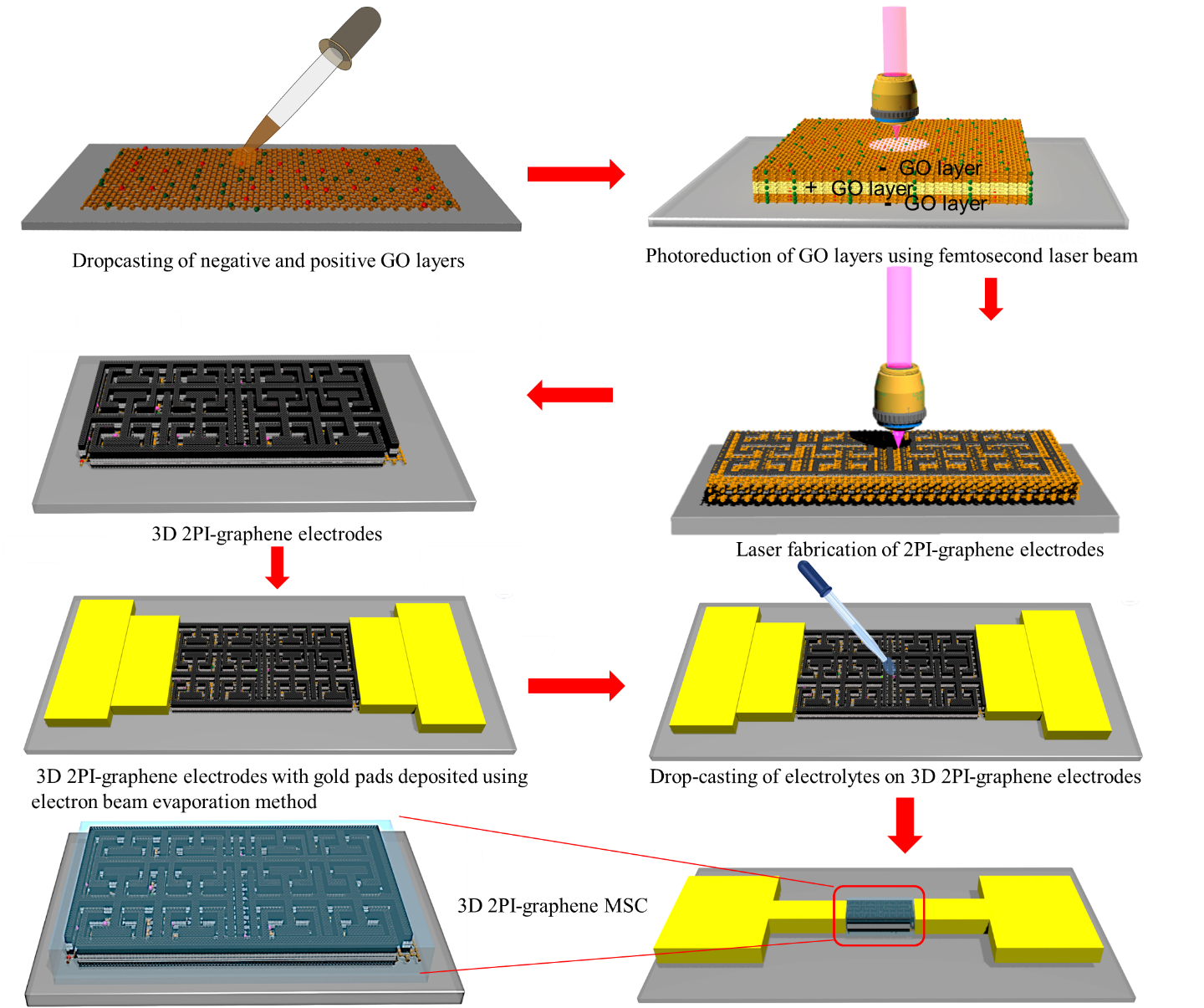
**

**Figure S1.** Schematic for the fabrication steps involved in the formation of 3D 2PI-graphene MSC.


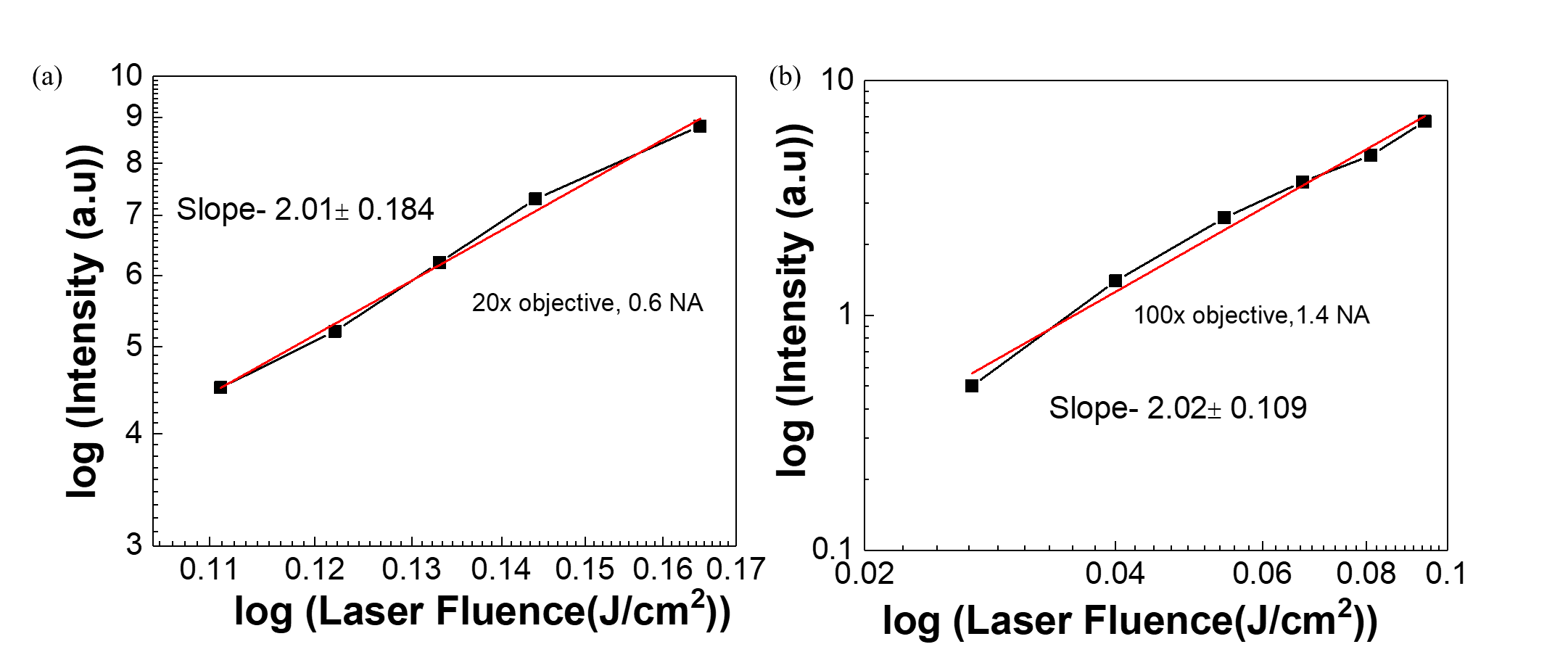


**Figure S2.** Two-photon reduction process in the single-layer graphene oxide film for the 80 MHz repetition rate at a wavelength of 800 nm with **(a)** a 20x air objective with 0.6 NA. **(b)** a 100x oil objective with 1.4 NA.


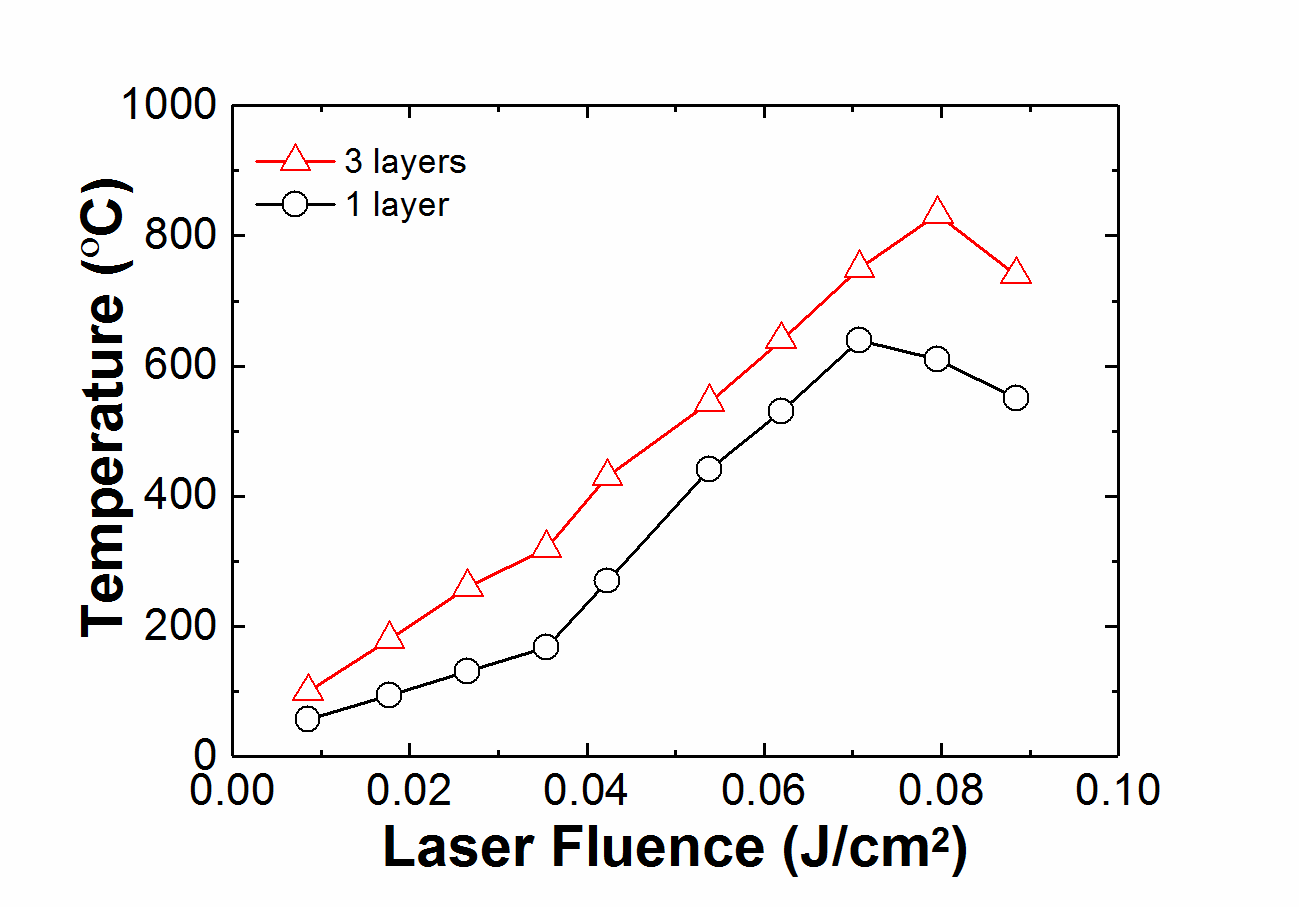


**Figure S3.** Simulated peak temperature profile of the single-layer graphene oxide film and three-layer graphene oxide films during the photoreduction using laser fluences at a wavelength of 800 nm and 80 MHz repetition rate.


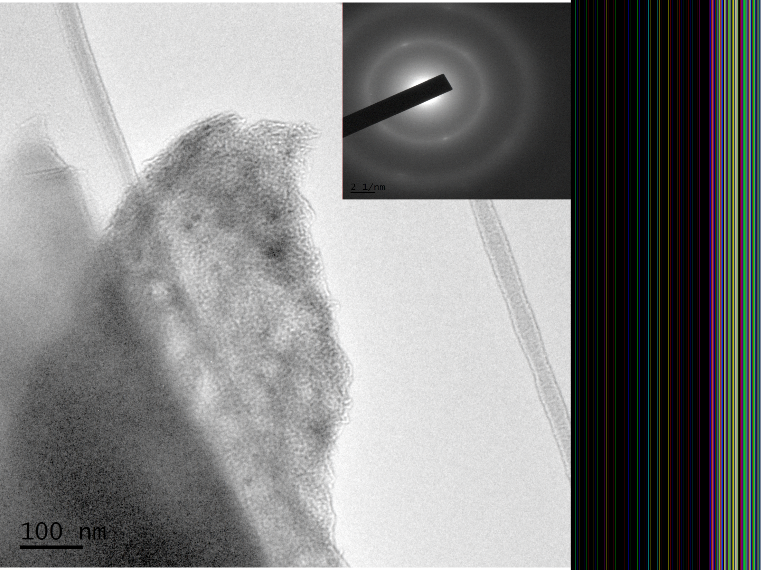


**Figure S4.** The image of transmission electron microscopy analysis for single- layer 2PI-graphene film during the photoreduction at a wavelength of 800 nm, 80 MHz repetition rate and a 100x oil objective with 1.4 NA.

**
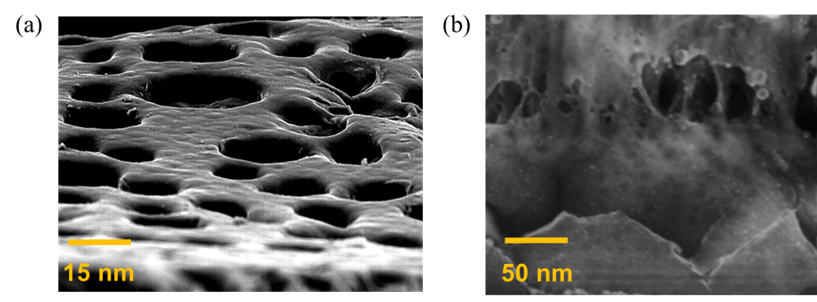
**

**Figure S5.** The porous morphology comparison for the photoreduced single-layer graphene oxide film for different repetition rates at a wavelength of 800 nm and a 100x oil objective with 1.4 NA. **(a)** 80 MHz. **(b)** 10 kHz (45° view).


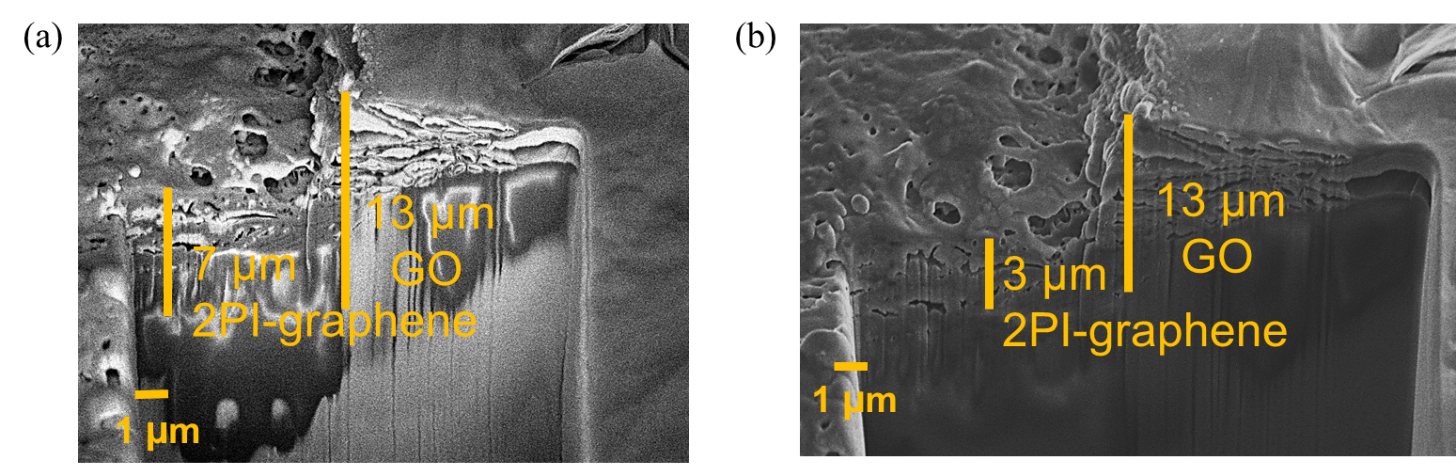


F**igure S6.** The scanning electron microscopy images for the thickness reduction obtained during the photoreduction on the single-layer graphene oxide film of thickness 13 µm at a wavelength of 800 nm and 80 MHz repetition rate **(a)** with a 20x air objective and **(b)** with a 100x oil objective (45° view).


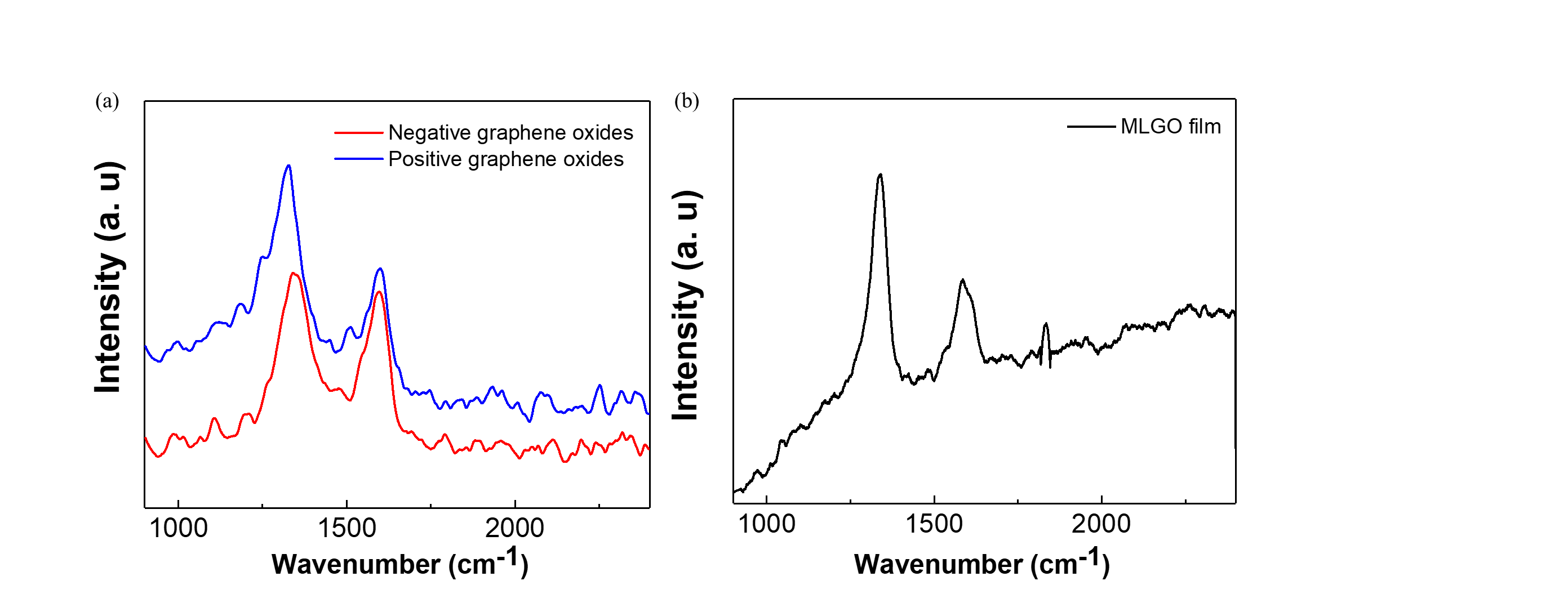


**Figure S7. Raman spectra of graphene oxide layers. (a)** Raman spectra of the negative and positive graphene oxide film. **(b)** Raman spectra of the MLGO film.


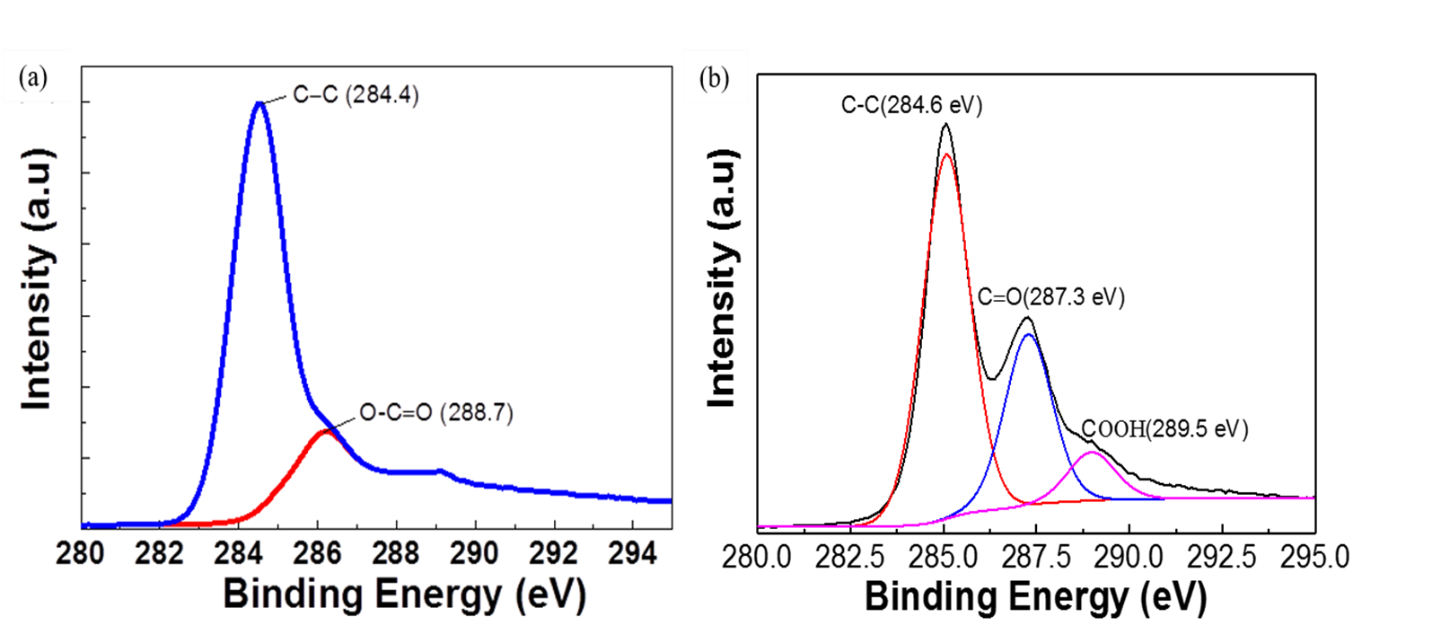


**Figure S8.** X-ray photoelectron spectroscopy (XPS) spectra of the 2PI-graphene film obtained at a wavelength of 800 nm with a 100x oil objective of 1.4 NA. **(a)** 80 MHz repetition rate. **(b)** 10 kHz repetition rate.


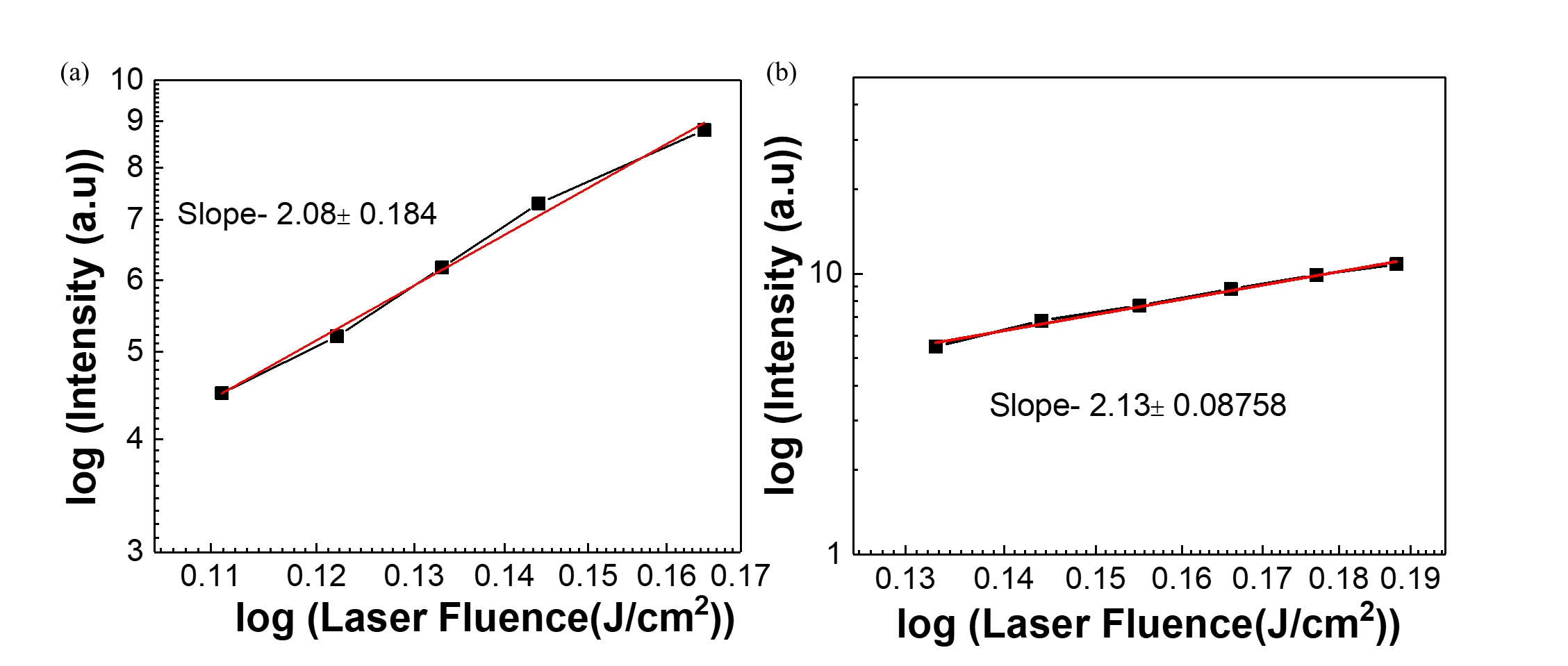


**Figure S9.** Logarithmic plot of laser fluence vs. intensity for two-photon absorption at a wavelength of 800 nm in **(a)** 2D graphene oxide film and **(b)** 3D graphene oxide film at the repetition rate of 80 MHz.


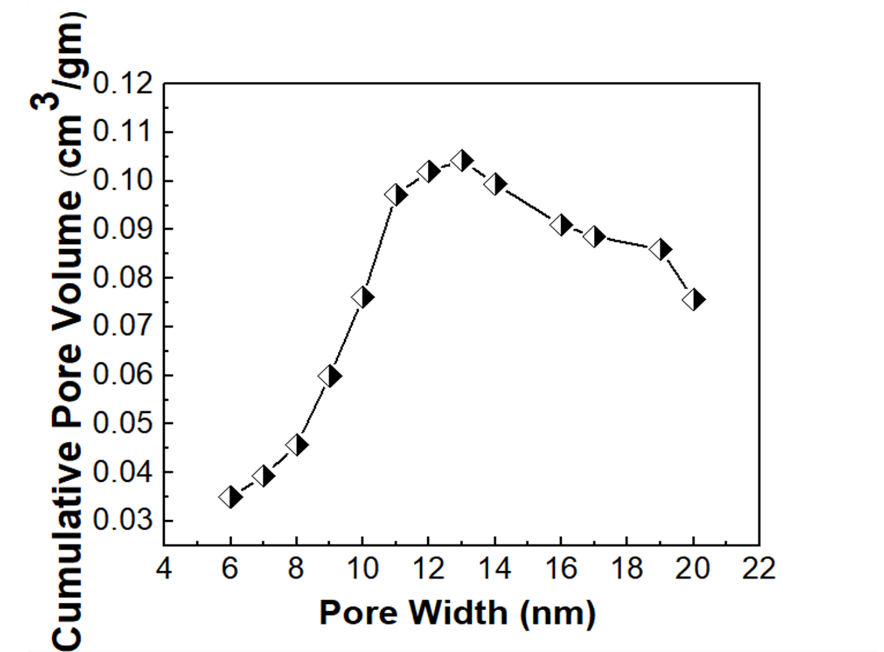


**Figure S10.** Porous analysis of 3D 2PI-graphene film obtained from the BJH method.

**Figure S11.** SAXS data for 2PI-graphene film.


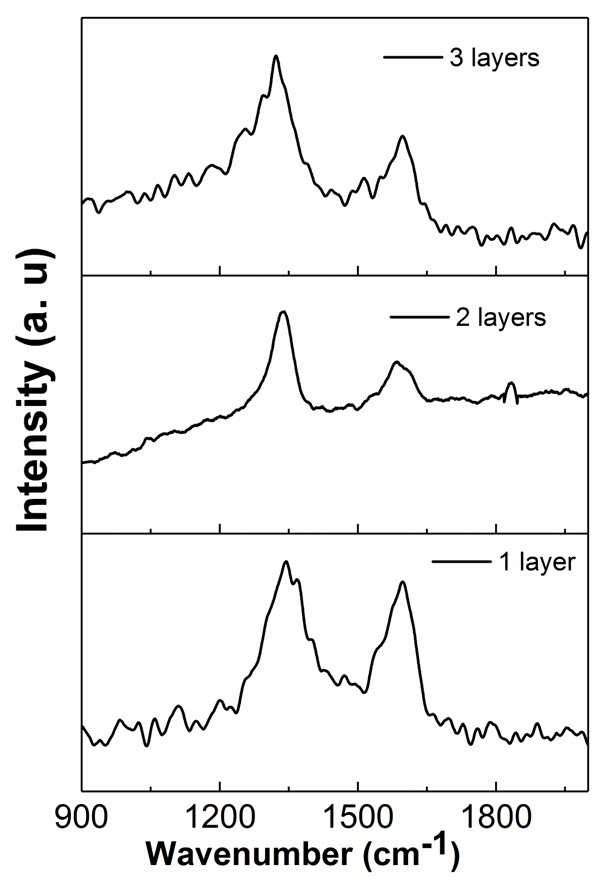


**Figure S12.** Raman spectra of various layers of the photoreduced graphene oxide film at a repetition rate of 80 MHz using a 20x objective with 0.6 NA.


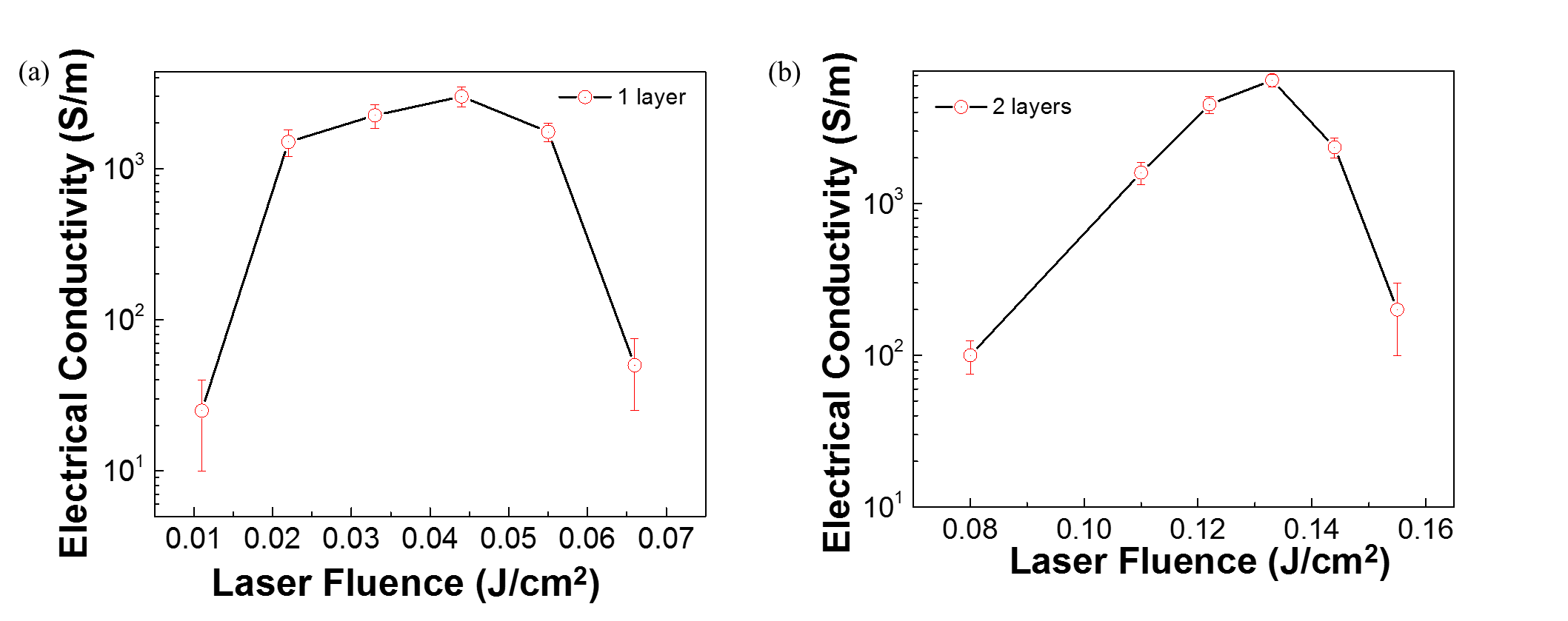


**Figure S13.** Electrical conductivity of a single-layer **(a)** and two-layers **(b)** photoreduced graphene oxide film at a wavelength of 800 nm and 80 MHz repetition rate using a 20x objective with 0.6 NA.


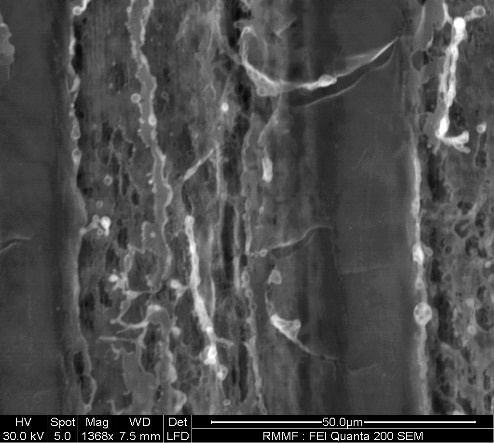


**1µm**

**1µm**

**LSGs**

**GOs**

**4 µm**

**Figure S14.** Cross-section SEM image of 3D 2PI-graphene patterns fabricated at the 80 MHz repetition rate at a wavelength of 800 nm using a 20x objective with 0.6 NA.


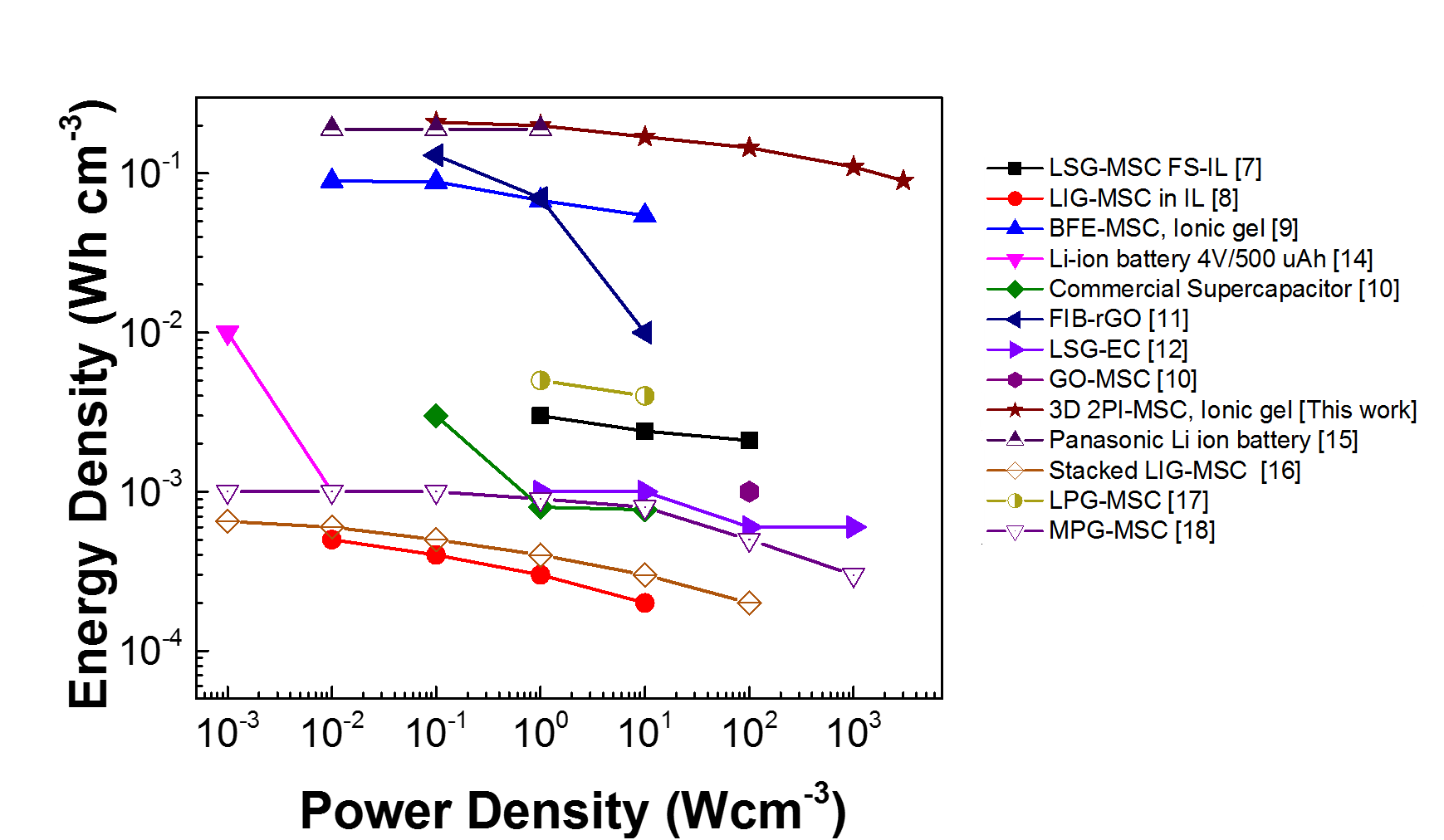


**Figure S15.** Ragone plot for the performance comparison between the supercapacitors made from lithographically induced graphene electrodes, commercial supercapacitors and batteries.

**Figure S16.** Ragone plot for the performance comparison of various lithographically induced based on areal capacitance.


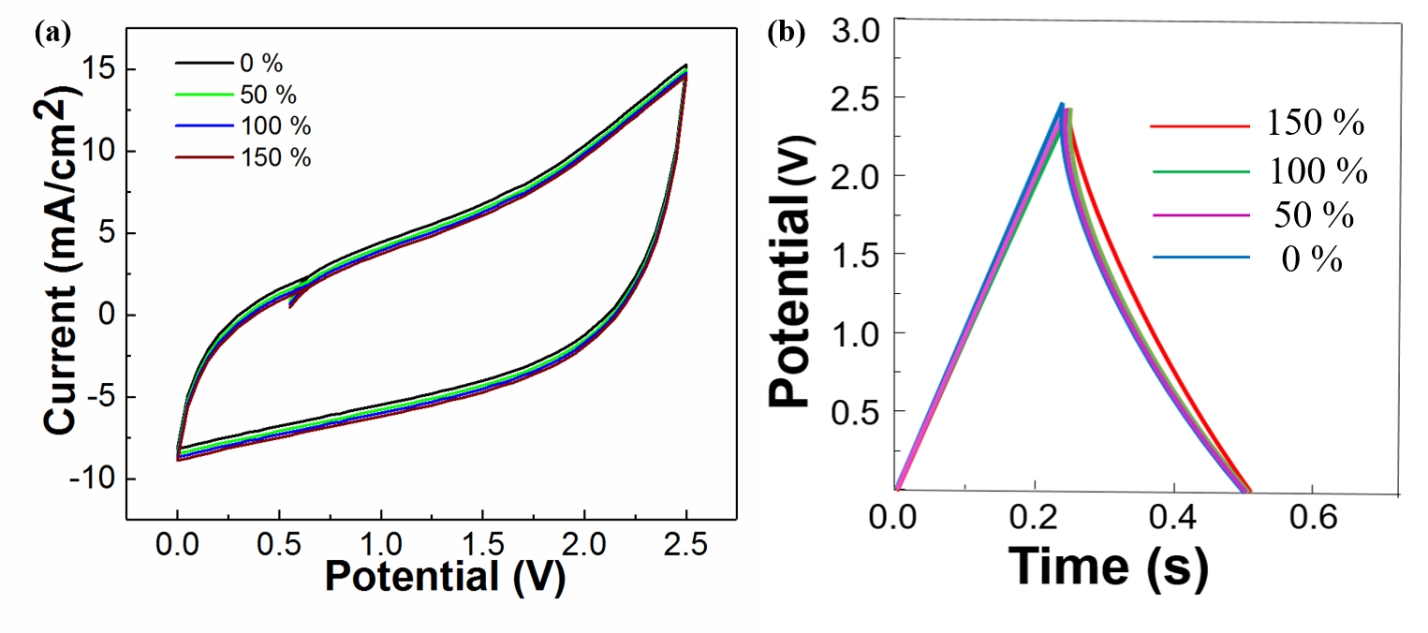


**Figure S17. Electrochemical characterizations on a stretchable 3D 2PI-graphene MSC. (a)** CV curves of the 3D 2PI-graphene MSC at a scan rate of 1000 mV s^-1^ at different stretchable distances. **(b)** Galvanostatic charge-discharge (CC) curves of the 3D 2PI-graphene MSC at various stretchable distances with a current density of 10 mA cm^-2^. All measurements provided are an average of 10 measurements.

**Table S1.** Properties of single-layer graphene oxide and 2PI-graphene films obtained for the repetition rate of 80 MHz at a wavelength of 800 nm with a 100x oil objective under the optimum laser fluence of 0.18 J/cm^2^.

| Properties | Graphene oxides | 2PI-graphene |
| --- | --- | --- |
| I_D_/I_G_ | 0.8 | 0.91 |
| sp^2^/sp^3^ | 0.98 | 1.6 |
|  |  |  |

**Table S2.** Comparison between the performances of single-layer and 2D 2PI-graphene MSCs.

| Types | Volumetric capacitance  (mF/cm^3^) at a scan rate of 3000 mVs^-1^ | Areal capacitance  (mF/cm^2^) at a scan rate of 3000 mVs^-1^ |
| --- | --- | --- |
| Single-layer negative graphene oxide film | 28 | 0.064 |
| Single-layer positive graphene oxide film | 34 | 0.078 |
| Two-layer of alternate layers of positive and negative graphene oxide film | 60 | 0.138 |

**Table S3.** Comparison between the stretchability and the volumetric capacitance of the 3D LSG supercapacitor ^[13]^ and the 3D 2PI-graphene MSC (this work).

| Type | Stretchability (%) | Volumetric capacitance  (mFcm^-3^) |
| --- | --- | --- |
| Transferred  Stretchable LSG  supercapacitor^[13]^ | 50 | 0.65 |
| This work | 150 | 86 |

**References**

1. Li, J-L. *et al.* Graphene oxide nanoparticles as a nonbleaching optical probe for two- photon luminescence imaging and cell therapy. *Angew. Chem. In. Ed.* **51**, 1830-1834 (2012).
2. Zhang, Y. L. *et al.* Photoreduction of graphene oxides: methods, properties, and applications. *Adv. Opt. Mater.* **2**, 10-28 (2014).
3. Zhou, Y. *et al.* Microstructuring of graphene oxide nanosheets using direct laser writing. *Adv. Mater.* **22**, 67-71 (2010).
4. Liska, R. & Ovsianikov, A. Multiphoton Lithography: Techniques, Materials, and Applications. *John Wiley & Sons, Weinheim* (2016).
5. Pei, S. & Cheng, H.-M. The reduction of graphene oxide. *Carbon* **50**, 3210-3228 (2012).
6. Cancado, L. *et al.* General equation for the determination of the crystallite size La of

nanographite by Raman spectroscopy. *Appl. Phys. Letts* **88**, 163106 (2006).

1. El-Kady, M. F. & Kaner, R. B. Scalable fabrication of high-power graphene micro-supercapacitors for flexible and on-chip energy storage. *Nat. Commun.* **4**, 1475 (2013).
2. Lin, J. *et al.* Laser-induced porous graphene films from commercial polymers. *Nat. Commun.* **5,** 5714 (2014).
3. Thekkekara, L. V. & Gu, M. Bioinspired fractal electrodes for solar energy storages. *Sci Rep* **7,** 45585 (2017).
4. Gao, W. *et al.* Direct laser writing of micro-supercapacitors on hydrated graphite oxide films. *Nat. Nanotech.* **6**, 496-500, (2011).
5. Lobo, D. E., Banerjee, P. C., Easton, C. D. & Majumder, M. Miniaturized supercapacitors: Focused ion beam reduced graphene oxide supercapacitors with enhanced performance metrics. *Adv. Ener. Mater* **5,**  1500665 (2015).
6. El-Kady, M. F., Strong, V., Dubin, S. & Kaner, R. B. Laser scribing of high-performance and flexible graphene-based electrochemical capacitors. *Science* **335**, 1326-1330 (2012).
7. Lamberti, A., Clerici, F., Fontana, M. & Scaltrito, L. A Highly stretchable supercapacitor using laser induced graphene electrodes onto elastomeric substrate. *Adv. Ener. Mater.* **6,** 1600050 (2016).
8. Pech, D., Brunet, M., Durou, H., Huang, P., Mochalin, V., Gogotsi, Y., Taberna, P. L., Simon, P. Ultrahigh-power micrometre-sized supercapacitors based on onion-like carbon. *Nat. Nanotech.* ***5***, 651-654 (2010).
9. <https://industrial.panasonic.com/ww/products/batteries/secondary-batteries/lithium-ion/cylindrical-type/NCR18650PF> (2018).
10. Z. Peng, J. Lin, R. Ye, E. L. Samuel, J. M. Tour. Flexible and stackable laser-induced graphene supercapacitors. *ACS applied materials & interfaces* **28**; 7(5):3414-9 (2015).
11. B. Xie, Y. Wang, W. Lai, W. Lin, Z. Lin, Z. Zhang, F. Kang. Laser-processed graphene based micro-supercapacitors for ultrathin, rollable, compact and designable energy storage components. *Nano Energy* **26**, 276-285 (2016).
12. Z. S. Wu, K. Parvez, X. Feng, & K. Müllen. Graphene-based in-plane micro-supercapacitors with high power and energy densities. *Nat. Commun.* **4**, 2487 (2013).
13. A. Lamberti, F. Perrucci, M. Caprioli, M. Serrapede, M. Fontana, S. Bianco, S. Ferrero, and E. Tresso. New insights on laser-induced graphene electrodes for flexible supercapacitors: tunable morphology and physical properties. *Nanotechnology*, *28*(17), p.174002 (2017).
